# A machine learning approach to predict mortality and neonatal persistent pulmonary hypertension in newborns with congenital diaphragmatic hernia. A retrospective observational cohort study

**DOI:** 10.1007/s00431-025-06073-0

**Published:** 2025-03-11

**Authors:** Luana Conte, Ilaria Amodeo, Giorgio De Nunzio, Genny Raffaeli, Irene Borzani, Nicola Persico, Alice Griggio, Giuseppe Como, Mariarosa Colnaghi, Monica Fumagalli, Donato Cascio, Giacomo Cavallaro

**Affiliations:** 1 Department of Physics and Chemistry, Università Degli Studi Di Palermo, Palermo, Italy; 2 Laboratory of Biomedical Physics and Environment, Department of Mathematics and Physics “E. De Giorgi”, University of Salento, Lecce, Italy; 3 Advanced Data Analysis in Medicine (ADAM), Laboratory of Interdisciplinary Research Applied to Medicine (DReAM), Local Health Authority (ASL) Lecce, Lecce, Italy; 4 Neonatal Intensive Care Unit, Fondazione IRCCS Ca’ Granda Ospedale Maggiore Policlinico, Milan, Italy; 5 Pediatric Radiology Unit, Fondazione IRCCS Ca’ Granda Ospedale Maggiore Policlinico, Milan, Italy; 6 Prenatal Diagnosis and Fetal Surgery Unit, Fondazione IRCCS Ca’ Granda Ospedale Maggiore Policlinico, Milan, Italy; 7 Department of Clinical Sciences and Community Health, Università Degli Studi Di Milano, Milan, Italy; 8 Department of Obstetrics and Gynecology, ASST Fatebenefratelli Sacco, Ospedale Macedonio Melloni, Milan, Italy

**Keywords:** Newborn, Congenital diaphragmatic hernia, Neonatal persistent pulmonary hypertension, Mortality, Machine learning, Deep learning

## Abstract

Congenital diaphragmatic hernia (CDH) has high morbidity and mortality rates. This study aimed to develop a machine learning (ML) algorithm to predict outcomes based on prenatal and early postnatal data. This retrospective observational cohort study involved infants with left-sided CDH, born from 2012 to 2020. We analyzed clinical and imaging data using three classification algorithms: XGBoost, Support Vector Machine, and *K*-Nearest Neighbors. Medical records of 165 pregnant women with CDH fetal diagnosis were reviewed. According to inclusion criteria, 50 infants with isolated left-sided CDH were enrolled. The mean o/eLHR was 37.32%, and the average gestational age at delivery was 36.5 weeks. Among these infants, 26 (52%) had severe persistent neonatal pulmonary hypertension (PPHN), while 24 (48%) had moderate or mild form; 37 survived (74%), and 13 did not (26%). The XGBoost model achieved 88% accuracy and 95% sensitivity for predicting mortality using ten features and 82% accuracy for PPHN severity with 14 features. The area under the ROC curve was 0.87 for mortality and 0.82 for PPHN severity.

*Conclusion:* ML models show promise in predicting CDH outcomes and supporting clinical decisions. Future research should focus on more extensive studies to refine these algorithms and improve care management.

*Clinical trial registration*: NCT04609163.

**Table:** 

**What is Known:** • *Congenital diaphragmatic hernia (CDH) is a serious condition characterized by high morbidity and mortality rates, making it critical to predict neonatal outcomes for effective clinical management accurately*. • *Traditional prenatal diagnostic methods often struggle to predict complications such as Neonatal Persistent Pulmonary Hypertension (PPHN) in CDH, highlighting the need for innovative predictive approaches*.	
**What is New:** • *Machine learning (ML) models, particularly XGBoost, have been shown to accurately forecast mortality and the severity of PPHN in infants with CDH based on prenatal and early postnatal clinical and imaging data*. • *ML-based predictive models can enhance prenatal counseling, optimize birth planning, and tailor postnatal care for patients with CDH, enabling real-time risk assessment and adaptive management strategies*.

**What is Known:**

• *Congenital diaphragmatic hernia (CDH) is a serious condition characterized by high morbidity and mortality rates, making it critical to predict neonatal outcomes for effective clinical management accurately*.

• *Traditional prenatal diagnostic methods often struggle to predict complications such as Neonatal Persistent Pulmonary Hypertension (PPHN) in CDH, highlighting the need for innovative predictive approaches*.

**What is New:**

• *Machine learning (ML) models, particularly XGBoost, have been shown to accurately forecast mortality and the severity of PPHN in infants with CDH based on prenatal and early postnatal clinical and imaging data*.

• *ML-based predictive models can enhance prenatal counseling, optimize birth planning, and tailor postnatal care for patients with CDH, enabling real-time risk assessment and adaptive management strategies*.

## Introduction

Congenital diaphragmatic hernia (CDH) is a rare congenital anomaly characterized by incomplete closure of the diaphragm and herniation of abdominal organs into the chest, resulting in pulmonary hypoplasia, persistent neonatal pulmonary hypertension (PPHN), and cardiac dysfunction [1–3]. CDH occurs in nearly 1 in 2500 births. Several factors influence the prognosis, such as defect size and location, associated anomalies, presence of liver up in the thorax, and gestational age at birth [4, 5]. Risk stratification is essential to identify patients who might benefit from specific interventions and to enable a risk-adjusted analysis of outcomes, healthcare costs, and management approaches. Prenatal and postnatal CDH predicting tools have largely increased and have been validated during the last years based on clinical and instrumental data [6–18]. However, a universal risk stratification method has not been identified yet, and an agreed-upon set of risk-specific management guidelines is still lacking [19].

In particular, predicting the severity of PPHN using conventional prenatal diagnostic methods remains challenging. As a result, there is growing interest in leveraging advanced technologies that favor a timely and accurate prognosis.

Artificial Intelligence (AI) is increasingly applied in the neonatal field to support medical data analysis. Predictive algorithms are being developed using traditional Machine Learning (ML) approaches as well as its more advanced Deep Learning (DL) extension. ML and DL can process and analyze medical data, including images from different sources such as ultrasound, magnetic resonance imaging (MRI), and X-ray. Integrating these algorithms into healthcare systems holds promise for enhancing diagnostic accuracy and disease pattern classification. These algorithms could help predict specific outcomes, guide interventions, and improve the overall quality of care [20–28].

However, to our knowledge, these methodologies still need to be successfully applied to newborns with CDH. The aim of our study was to provide a predictive algorithm for mortality and PPHN in CDH based on the integrated analysis of prenatal and early postnatal data.

## Materials and methods

### Study design

This study represents an exploratory secondary analysis of a retrospective observational cohort study performed at Fondazione IRCCS Ca’ Granda Ospedale Maggiore Policlinico, Milan, Italy (CLANNISH, Clinical Trials identification no: NCT04609163) [20]. The study involved the following services: the Fetal Surgery Center, Pediatric Radiology Service, and Neonatal Intensive Care Unit (NICU). Moreover, the Department of Mathematics and Physics at the Università del Salento (Lecce, Italy) and the Department of Physics and Chemistry at the Università degli Studi di Palermo (Palermo, Italy) developed the AI algorithms.

The current study adhered to the principles of good clinical practice and followed the guidelines of the Helsinki Declaration. It received approval from the local ethics committee (Milan Area 2, Italy) with approval number/ID 800_2020bis and subsequent amendment 287_2021. However, considering its retrospective design, the ethics committee waived the need for informed consent. The study was also registered on ClinicalTrials.gov with the identifier NCT04609163.

### Patients

The study population, inclusion–exclusion criteria, and a comprehensive description of the primary study design were previously published and are briefly summarized here [20]. Inborn CDH patients born between 01/01/2012 and 31/12/2020 admitted to the NICU at birth were included. The mothers’ take-charge took place at our Fetal Surgery Center at a gestational age of 30 + 6 weeks or below. Non-isolated CDH and twin pregnancies were excluded. Only left-sided CDH were considered because of their larger numerosity, homogeneity, and variability in liver position, leaving out right-sided CDH.

### Data collection

Clinical maternal and fetal prenatal variables were retrospectively collected using Astraia software (Astraia Software GmbH, Ismaning, Germany) and NeoCare software (GPI SpA, Trento, Italy). A prenatal ultrasound (US) performed between 25 + 0 and 30 + 6 weeks of gestation was considered for each patient. In the case of fetal endoscopic tracheal occlusion (FETO), the fetal US was performed before the fetal procedure. Additionally, native sequences from fetal MRI were gathered for 37 out of 50 cases, with separate acquisitions for the lung and liver. The imaging software employed for this study was Synapse PACS and Synapse 3D (FUJIFILM Medical Systems, Lexington, MA, US). Lung volumes were computed using T2 HASTE sequences, selecting the best-quality image plane without motion-induced artifacts [29]. On the other hand, liver volumes were calculated based on T1 VIBE sequences [30]. An experienced pediatric radiologist (I. B.) freehand delineated Regions of Interest (ROIs) to define the areas of the left and right lungs and the liver, excluding the pulmonary hila and mediastinal structures for each slice. Organ volumes were calculated using the software. Subsequently, the DICOM (Digital Imaging and Communications in Medicine) files were anonymized and converted to the neuroimaging informatics technology initiative (NIfTI) format for easy manipulation.

### Clinical and imaging variables

A detailed summary of the clinical and imaging variables included is reported in Table 1.

**Table 1:** Clinical and imaging variables

***Maternal data***
Mother age
Ethnicity
Number of gestations ending the outcome for fetuses with assisted reproduction (MAR): yes/no
Antenatal use of corticosteroids: yes/no
Premature rupture of membranes (pPROM): yes/no
Gestational age at pPROM
Gestational age at CDH diagnosis
***Fetal ultrasound data***
Gestational age
Estimated fetal weight (EFW)
Amniotic fluid (AF)
Umbilical artery pulsatility index
Pulmonary flow pulsatility index
Pulmonary flow peak systolic velocity
Peak early diastolic reversed flow
Organ herniation of bowel, stomach, liver: yes/no
Observed/expected lung-to-head ratio (tracing method)
CDH severity: mild, moderate, severe
Fetal endoscopic tracheal occlusion (FETO): yes/no
Gestational age at balloon insertion and removal
***MRI data***
Gestational age
Apparent diffusion coefficient (ADC)
Right and left lung volume
Total fetal lung volume (TFLV)
Observed/expected TFLV (O/E TFLV%)
Total liver volume
Herniated liver volume
Percentage of liver herniation (%LH)
Mediastinal shift angle (MSA)
Apparent diffusion coefficient (ADC) of the left and right lung
***Early postnatal data***
Gestational age at birth
Birth weight

### Radiomics features

In addition to the clinical variables, standard 3D radiomics features were extracted from the segmented ROIs in the MRI using the freely available and open-source Pyradiomics v. 3.01 software tool [22, 29–31]. Pyradiomics produces many variables, with and without preprocessing by various filters and optional reslicing, with different interpolators. Only features from the original images without preprocessing were considered in this work. Due to significant dissimilarity in the gray-level content of the MRI scans, only shape features were utilized (MeshVolume, VoxelVolume, SurfaceArea, SurfaceVolumeRatio, Sphericity, Maximum3DDiameter, MajorAxisLength, MinorAxisLength, LeastAxisLength, Elongation, and Flatness). The geometric meaning of each feature is detailed in Table 2.

**Table 2:** Geometric meaning of Pyradiomics shape features

*Feature*	*Geometric meaning*
MeshVolume	The shape volume is calculated using a mesh representation. It is the three-dimensional space enclosed by the surface of the object
VoxelVolume	The volume is calculated by counting the number of voxels (3D pixels) within the shape and multiplying by the volume of a single voxel. This represents the discretized volume of the object
SurfaceArea	The total area of the surface of the shape. This quantifies the two-dimensional extent of the object’s surface
SurfaceVolumeRatio	The ratio of surface area to volume. This measure indicates how “compact” an object is; lower values suggest a more compact shape
Sphericity	A measure of how spherical (round) the object is. Perfect spheres have a sphericity of 1. Lower values indicate less spherical shapes
Maximum3DDiameter	The largest distance between any two points on the surface of the shape. This is the maximum length of the object in any dimension
MajorAxisLength	The length of the major axis of the shape, which is the longest dimension in the principal component analysis (PCA) of the object
MinorAxisLength	The length of the minor axis, which is perpendicular to the major axis and is the second longest dimension in the PCA of the object
LeastAxisLength	The shortest axis length from the PCA of the object. It is perpendicular to both the major and minor axes
Elongation	The ratio of the minor axis length to the major axis length. It indicates how much longer the shape is in one direction compared to the other
Flatness	The ratio of the least axis length to the major axis length. This measure indicates how “flat” or “elongated” an object is compared to being spherical

Variables calculated from the gray levels were discarded to avoid additional image manipulation, such as intensity standardization. A total of 80 features were considered, including 56 prenatal variables, two very early postnatal variables (gestational age and birth weight), and 22 MRI-extracted shape features (11 from the lungs and 11 from the liver).

To ensure fairness in the classification process, the features were normalized to the 0–1 range using min–max normalization on the training set. The same normalization parameters were then applied to the validation set samples. However, in some cases, the lack of MRI data resulted in missing values in the features extracted by Pyradiomics. This was also observed for non-radiomics features based on the patient’s diagnostic pathway. Imputation by a weighted average was considered to handle these missing values, as in Eq. 1 [32]:

1 $${f}_{n}\left(m\right)=\frac{\frac{\overline{{f }_{n}^{\left(1\right)}}}{{\sigma }_{n}^{\left(1\right)}}+\frac{\overline{{f }_{n}^{\left(2\right)}}}{{\sigma }_{n}^{\left(2\right)}}}{\frac{1}{{\sigma }_{n}^{\left(1\right)}}+\frac{1}{{\sigma }_{n}^{\left(2\right)}}}$$

where ${f}_{n}\left(m\right)$ is the value to be assigned to the (missing) *n*-th feature for the *m*-th sample, $\overline{{f }_{n}^{\left(1\right)}}$ and $\overline{{f }_{n}^{\left(2\right)}}$ are the average values of the *n*-th features for classes 1 and 2, respectively, and ${\sigma }_{n}^{\left(1\right)}$ and ${\sigma }_{n}^{\left(2\right)}$ are the corresponding standard deviations. This way, in the approximation of Gaussian distributions, a neutral value for the distributions of the two classes is used as the missing feature.

### Target variables: neonatal persistent pulmonary hypertension and mortality

Echocardiograms were performed by only two pediatric cardiologists with Canon Aplio i700 (Canon Medical Systems Corporation, Otawara-shi, Japan). For each included patient, a neonatologist performed a systematic revision of the first available echocardiogram within 24 h after birth, focusing on direct and indirect signs of PPHN. PPHN was defined as persistently elevated pulmonary vascular resistance, resulting in a significant decrease in pulmonary blood flow [33]. Data collection was focused on the presence and characteristics of the shunts through patent *ductus arteriosus* and *foramen ovale*, the characteristics of the intraventricular sept, the estimation of the systolic pulmonary artery pressure through tricuspid valve regurgitation, the systemic pressures, and concomitant use of pulmonary vasodilators. Patients were then stratified according to the presence and severity of pulmonary hypertension into two categories: severe (over-systemic, considered as the positive class) vs moderate/mild (iso/under-systemic). According to mortality, the study population was divided into non-survivors (positive class) *vs* survivors.

### Feature selection

We employed the Recursive Feature Elimination (RFE) technique with Cross-Validation (CV), specifically using the Leave One Patient Out CV (LOPO-CV) scheme. The RFE method starts with the entire feature set and recursively removes the minor essential features based on a chosen metric (in this case, accuracy) until the desired number of features is reached. Typically, the final number of features to select is a parameter that needs to be specified. This parameter was determined dynamically by varying its value and calculating the corresponding accuracy in our approach. We then selected the parameter value that maximized accuracy. The Random Forest (RF) classifier evaluated the various configurations.

### Training

To optimize the utilization of available samples, we implemented a LOPO-CV strategy. In this methodology, one patient was selected as the validation set, while the remaining patients were employed for training purposes. We trained multiple classifiers and evaluated their performance using various metrics, including the confusion matrix, sensitivity, specificity, the area under the Receiver Operating Characteristic (ROC) curve (AUC), and the area under the Precision-Recall (P-R) curve. All optimization procedures were conducted with the primary objective of maximizing accuracy.

### Classifiers

Three classification algorithms were tested: eXtreme Gradient Boosting (XGBoost), Support Vector Machine (SVM), and K-Nearest Neighbors (KNN). The first classifier was used because it natively and effectively deals with missing clinical values. The choice of the other two classifiers was due to their ability to allow good performance in conditions of a limited number of available samples, as they are characterized by a reduced number of parameters to be tuned [34]. Hyperparameter tuning was performed to avoid overfitting and improve model performance.

## Results

**Fig. 1:**
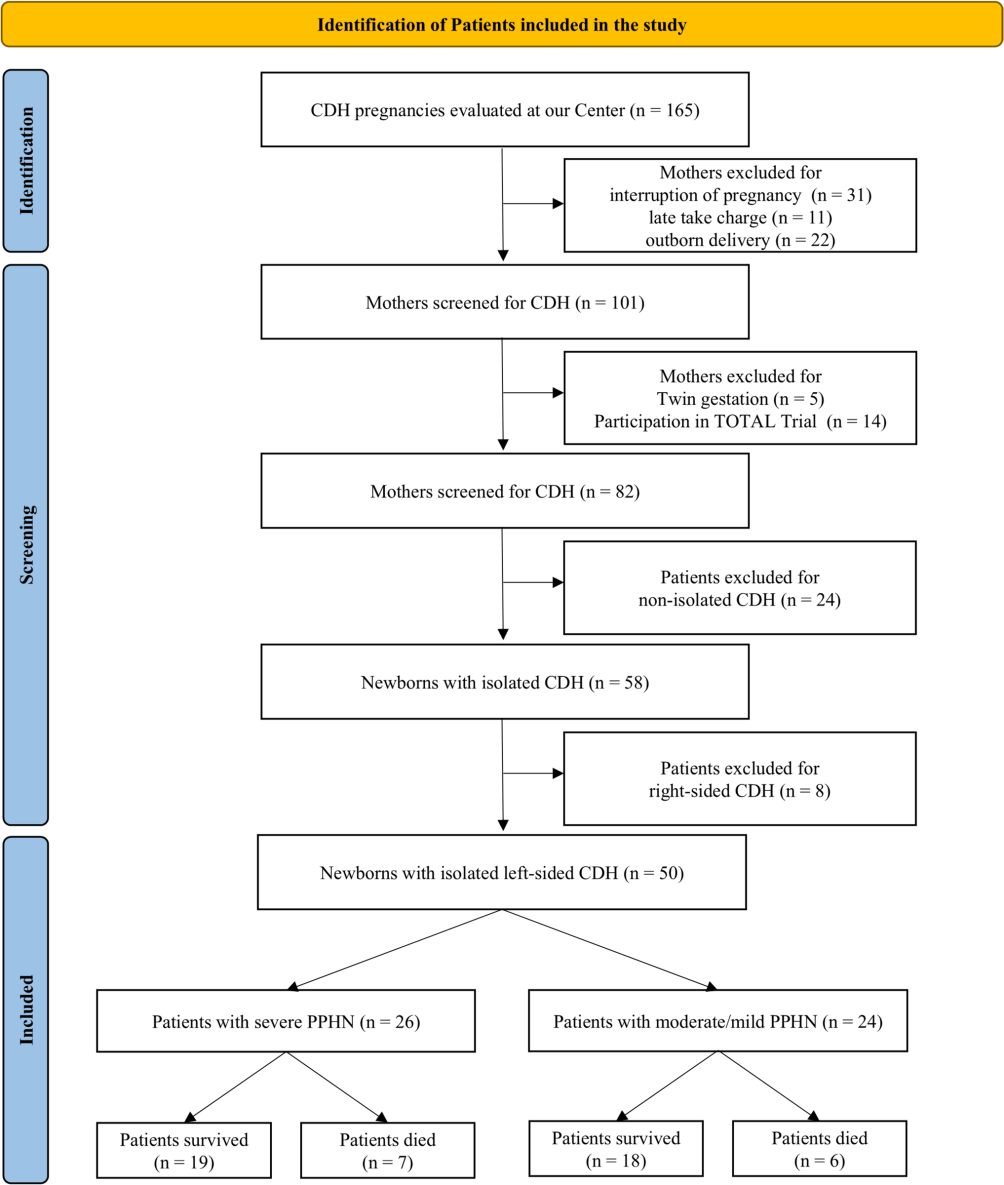
The PRISMA flow diagram of the study

Figure 1 presents the flow diagram of the study [35]. The medical records of 165 pregnant women referred to our Center following a prenatal diagnosis of CDH during the specified years were reviewed. Of these, 101 women were screened for the study, while the others were excluded for subsequent interruption of pregnancy, outborn delivery, or late take charge. In addition, five were excluded for twin gestation and 14 for participation in the TOTAL trial [36]. Following the pre- or postnatal definition of non-isolated disease, 58 newborns were classified as having isolated CDH. Out of these, eight patients were excluded for right-sided defects. In total, 50 eligible infants were included in the analysis: 26 were classified as severe (52%) and 24 as moderate/mild (48%) cases of PPHN. Regarding mortality, there were 37 survivors (74%) and 13 non-survivors (26%) (Fig. 1). Table 3 provides a summary of the demographic characteristics of the population under study.

**Table 3:** Demographic characteristics

Demographic data	Isolated left-sided CDH (*n* = 50)
Gestational age, weeks, mean (SD)	36.5 (2.5)
Birthweight, grams, mean (SD)	2786.8 (563.9)
Male, *n* (%)	27 (54)
Gestational age at diagnosis of CDH, mean (SD)	20.8 (3)
Severe CDH, *n* (%)	16 (32)
FETO, *n* (%)	14 (28)
o/e LHR, %, mean (SD)	37.32 (11.9)
o/e TFLV, %, mean (SD) (*n* = 37)	35.6 (12.9)
MSA, degrees, mean (SD) (*n* = 37)	37 (5.5)
LH, %, mean (SD) (*n* = 37)	10.1 (15.3)
Preterm premature rupture of membranes, *n* (%)	19 (38)
Antenatal steroids, *n* (%)	16 (32)
Cesarean section, *n* (%)	22 (44)
Apgar 1st minute, median [IQR]	6 (4–7)
Apgar 5th minutes, median [IQR]	8 (8–9)
Days of mechanical ventilation, mean (SD)	17.9 (7.2)
Severe PPHN, *n* (%)	26 (52)
Death, *n* (%)	13 (26)

*CDH* congenital diaphragmatic hernia, *FETO* fetal endoscopic tracheal occlusion, *IQR* interquartile range, *LH* liver herniation, *PPHN* neonatal persistent pulmonary hypertension, *o/e LHR* observed/expected lung-to-head ratio, *o/e TFLV* observed/expected total fetal lung volume, *MSA* mediastinal shift angle, *n* number, *SD* standard deviation

As regards mortality analysis, the feature selection procedure led to the choice of 10 out of 80 features, in particular: maternal age, gestational age at CDH diagnosis, CDH severity (mild, moderate, severe), centile of estimated fetal weight (EFW), observed/expected lung to head ratio (o/e LHR) by tracing method, umbilical artery pulsatility index (AU-PI), left and right fetal lung volume (FLV) at MRI, observed/expected total fetal lung volume (o/e TFLV), gestational age at birth, and birth weight. Remarkably, no shape features were selected. Table 4 shows the classification results obtained for mortality prediction.

**Table 4:** Classification figures of merit for mortality, computed in a LOPO scheme

*Classifier*	*AUC*	*Accuracy*	*Sensitivity*	*Specificity*
XGBoost	0.87	88%	95%	69%
SVM	0.78	80%	97%	31%
KNN	0.78	82%	95%	46%

*AUC* area under the curve, *KNN k*-nearest neighbors, *LOPO* leave one patient out, *SVM* support vector machine, *XGBoost* extreme gradient boosting

**Fig. 2:**
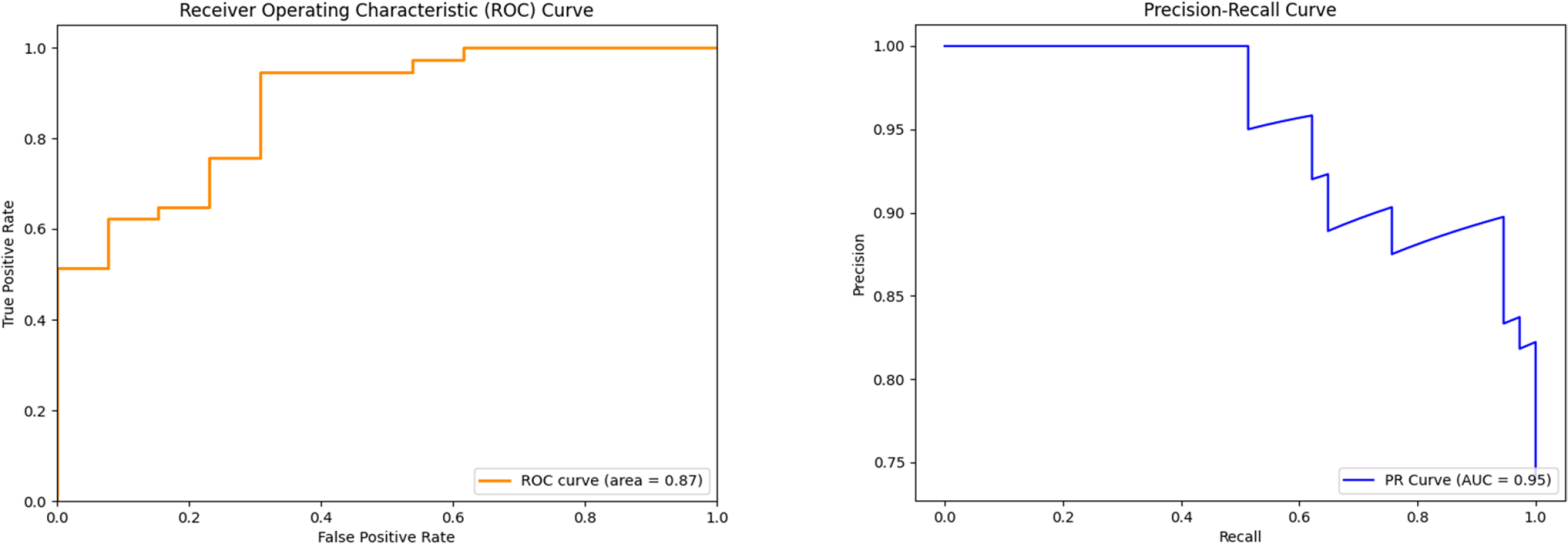
Mortality prediction. Left, Receiver Operating Characteristic (ROC) curve; right, the Precision-Recall (P-R) curve for mortality predictions, obtained with the XGBoost classifier on prenatal clinical variables. No shape features extracted from magnetic resonance imaging (MRI) were used, as required by the feature-selection procedure

The results of the different classification methods were comparable regarding AUC and accuracy, though XGboost had better performance. Figure 2 shows the ROC and P-R curves for mortality prediction obtained by XGboost with feature selection. The trained model correctly identified 88% of cases and achieved a sensitivity of 95% and a specificity of 69%. The AUC from the ROC curve was 0.87, while the P-R curve subtended an area of 0.95 (with the frequency of positive cases equal to 52%). From the P-R curve, precision drops after 50% sensitivity but remains more than 85% when sensitivity is 90%, and even if we require complete sensitivity, precision remains relatively high (around 80%).

Table 5 reports the classification results obtained for PPHN classification. In this case, both clinical and shape features were selected. The results produced by the different classification methods show that XGboost also performed better in this case.

**Table 5:** Classification figures of merit for PPHN, computed in a LOPO scheme

*Classifier*	*AUC*	*Accuracy*	*Sensitivity*	*Specificity*
XGBoost	0.82	82%	85%	79%
SVM	0.75	74%	79%	71%
KNN	0.65	54%	53%	33%

*AUC* area under the curve, *KNN k*-nearest neighbors, *LOPO* leave one patient out, *PPHN* neonatal persistent pulmonary hypertension, *SVM* support vector machine, *XGBoost* extreme gradient boosting

**Fig. 3:**
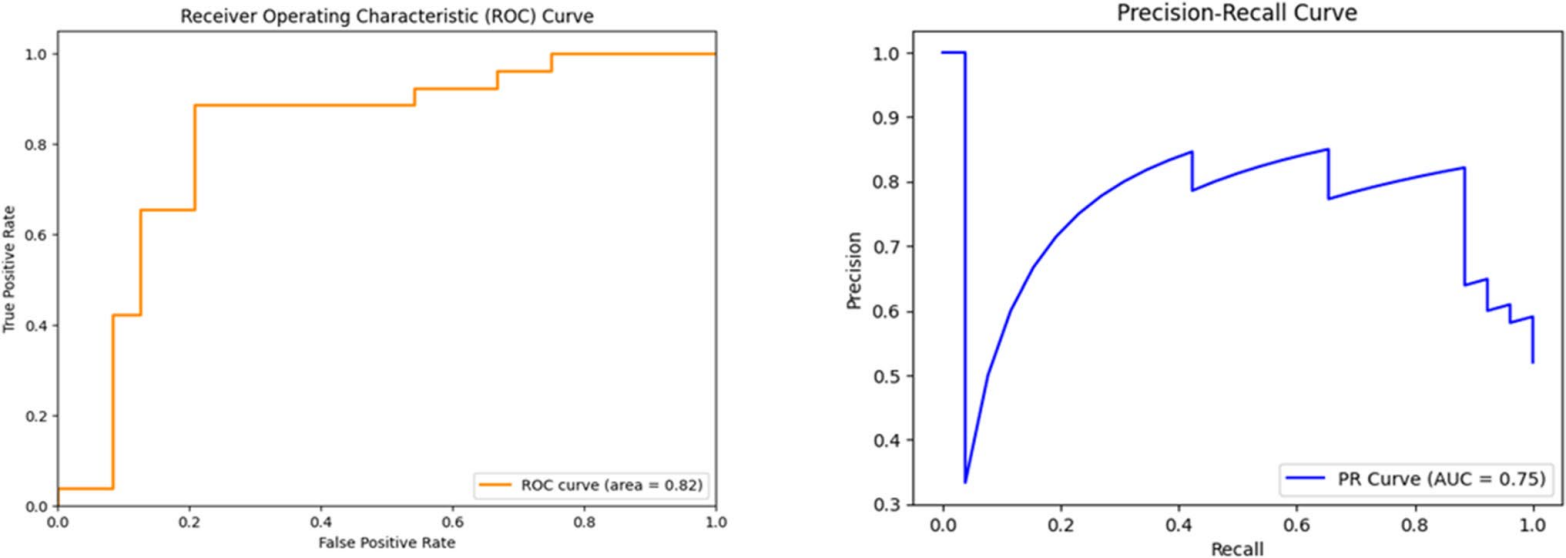
Neonatal persistent pulmonary hypertension (PPHN) prediction. Left, Receiver Operating Characteristic (ROC) curve; right, the Precision-Recall (P-R) curv**e** for PPHN predictions with the XGBoost classifier on prenatal clinical variables and shape features extracted from magnetic resonance imaging (MRI)

Figure 3 shows the ROC and P-R curves for PPHN classification obtained by XGboost with the features selection. The AUC from the test ROC curve was 0.82, while the P-R curve subtended an area of 0.75 (with the frequency of positive cases equal to 26%). The trained model correctly identified 82% of cases and achieved a sensitivity of 85% and a specificity of 79%. From the P-R curve, we deduce that even at very high sensitivity values (about 83–84%), precision is more than 80%.

## Discussion

CDH is a life-threatening anomaly requiring a highly skilled and multidisciplinary team of experts for appropriate management from the antenatal diagnosis [37].

Despite advancements over time, morbidity and mortality remain significant (20–40%), even within high-volume tertiary referral centers [38–40]. An estimated quarter of survivors experience neurodevelopmental impairments across all domains, encompassing motor and sensory (hearing, visual) deficits as well as cognitive, language, and behavioral impairments [41].

CDH patients exhibit varying degrees of pulmonary hypoplasia and abnormal pulmonary vascular disease, resulting in varying extents of pulmonary hypertension. Up to 30–40% of newborns with CDH experience concomitant cardiac ventricular dysfunction [42, 43]. PPHN is associated with adverse outcomes in CDH patients, underscoring the critical nature of its management in the care of these infants [44].

Various clinical and laboratory parameters and prognostic indices in the perinatal period have been subject to study to predict postnatal outcomes [39, 45–48]. Identifying variables predictive of mortality is paramount for clinical decision-making and parental guidance. The o/e LHR and o/e TFLV serve as pivotal metrics in this regard. Each parameter evaluates the extent of pulmonary hypoplasia associated with CDH, a critical determinant of survival and long-term prognosis.

The o/e LHR has been widely studied and utilized in predicting postnatal survival in cases of isolated CDH. Jani et al. [49] highlighted the significance of the o/e LHR in predicting survival in fetuses with isolated diaphragmatic hernia. Snoek et al. [50] further assessed the predictive value of the o/e LHR for survival and chronic lung disease (CLD) in survivors with left-sided CDH, reflecting its ongoing relevance in an era of standardized neonatal treatment. Their multicenter study underscores the evolving understanding of o/e LHR in predicting outcomes for CDH patients.

On the other hand, the o/e TFLV exhibits a stronger correlation with postnatal outcomes than the absolute lung volume. Moreover, a growing body of evidence supports the superior accuracy of o/e TFLV in predicting survival compared to ultrasound-based estimations of lung size, which may not fully account for the ipsilateral lung and could thus underestimate the effective lung volume [51–56].

In cases of isolated CDH, o/e TFLV has demonstrated efficacy in distinguishing survival, with an o/e TFLV < 25% being associated with more severe forms and a reduced survival rate [55, 57–61]. Furthermore, o/e TFLV has been shown to forecast the necessity for extracorporeal membrane oxygenation (ECMO) after birth, with the combined assessment of lung volumetry and o/e LHR proving more effective than ultrasound alone in predicting the need for ECMO [62–65].

Prenatal prediction of PPHN plays a crucial role in prenatal management, delivery planning, and postnatal care. However, while both o/e LHR and o/e TFLV offer insights into the extent of CDH-associated pulmonary hypoplasia, their predictive value for PPHN necessitates careful consideration.

Our findings support the possibility of successfully developing an ML system for predicting PPHN severity and mortality risk based on the integrated assessment of prenatal and early postnatal variables.

To achieve our goal, we enrolled 50 left-sided CDH cases. The dataset was relatively balanced concerning PPHN, with 26 severe and 24 moderate/mild cases, whereas mortality classes included 37 survivors and 13 non-survivors. We combined prenatal clinical and imaging data with gestational age and weight at birth, which both play a key role in survival in neonatal patients, especially those in critical conditions. In addition, standard 3D radiomics features were extracted from the segmented ROIs using the freely available Pyradiomics software tool. This software package facilitated automatic reslicing with a selected interpolator and computed multiple radiomics variables. As the MRIs exhibited significant variations in grayscales, which would have required some form of intensity standardization to use features based on gray values, we only utilized shape features to avoid additional image manipulation and discarded variables based on the gray levels.

A feature selection phase was executed for both postnatal target variables, mortality, and PPHN. The RFE approach used RF classification to evaluate different configurations, which was appropriate for several reasons. First, this approach provides features of relative importance during the training process. Each time a decision tree is constructed, the model tracks how much each feature contributes to reducing the cost function, usually Gini impurity or entropy. An importance score for each feature is obtained by averaging this importance across all trees. Second, because of its “forest” nature, an RF is robust and less prone to overfitting than individual decision trees. This means that the computed importance of features is more reliable and less affected by noise in the data. Finally, RFs can handle highly correlated features without special preprocessing. In the presence of correlations, this approach can distribute importance among correlated features, providing a complete picture of each feature contribution.

We comprehensively evaluated three classification algorithms: XGBoost, SVM, and KNN. Our models were trained using prenatal and early postnatal clinical variables, as well as selected shape features extracted from MRI data. Interestingly, we discovered that XGBoost outperformed the other models and emerged as the best classification model for both clinical targets. The supervised ML models, designed to predict PPHN severity and neonatal mortality, showed promising preliminary results. Our study suggests that predicting mortality and PPHN severity in the prenatal and very early postnatal period can be feasible by ML applications, achieving accuracies of 88% for mortality and 82% for postnatal PPHN. With significant accuracy rates and reliable sensitivity, this model has the potential to revolutionize prognostic assessment in CDH, eventually improving patient outcomes. By implementing the algorithm, risk categories could be simulated based on available prenatal data and assuming gestational age and estimated fetal weight at birth. The algorithm could also be updated in real-time at subsequent obstetric visits or based on the threat of preterm delivery, as prematurity plays a significant role in survival, especially in infants with underlying diseases. This would assist with parenting counseling, birth planning, and postnatal care. To the best of our knowledge, our studies are the first to explore the application of AI methods to CDH [20, 22].

Despite the encouraging data, some limitations must be considered. First, the restricted dataset deriving from the rarity of the condition represented the weakest point, as the relatively small sample size of 50 cases may limit the generalizability of our findings. The rarity of CDH poses constraints on data expansion, which could affect the robustness of our ML models. An appropriate number of cases during training/validation and data interpretation is crucial for ML applications. Therefore, more extensive multicenter studies are needed to validate these models and enhance their applicability across different clinical settings.

Additionally, this study’s retrospective nature introduces inherent limitations related to data collection, including potential biases and missing values. Although we implemented rigorous methods for handling missing data, such as weighted imputation techniques, some bias is still likely to persist. Future research would benefit from standardized prospective data collection to help address these issues.

Another critical aspect is data inhomogeneity, specifically the lack of a standard grayscale in the images. This would require a standardization procedure, after which gray-level-based features could increase ML quality for classification purposes. Nonetheless, MRI standardization is a delicate process that involves profound changes in image gray levels, which might even make ML procedures less accurate. Consequently, we preferred to discard ML features based on the gray-level content of ROIs, only using shape features. The exclusion of grayscale-based radiomic features from our MRI analysis, while reducing the complexities of image standardization and ensuring consistency in feature extraction, may have reduced the predictive power of the imaging parameters. Interestingly, no shape features were selected for the mortality target, whereas clinical and shape variables were selected for the PPHN target. We can speculate that the information provided by the images is more closely related to the structure and architecture of the lung parenchyma, which directly impacts the disease’s pathophysiology.

On the other hand, mortality may be an indirect result of these structural alterations, influenced by many factors. Although a conclusive interpretation is not yet possible, this aspect deserves further investigation, and an increase in the study population and image optimization are crucial. Future studies should investigate methods for standardizing grayscale intensity to allow the inclusion of a broader range of radiomic features.

Finally, the models require external validation on independent datasets to confirm their clinical applicability. Validation across multiple institutions is essential to ensure that the ML algorithms maintain predictive accuracy across diverse patient populations and imaging protocols. Collaborative efforts in data sharing and establishing standardized imaging methodologies will be critical for further refining these predictive models.

## Conclusions

Although with limitations, with reasonable accuracy, an ML approach for predicting mortality and PPHN severity of CDH newborns using prenatal and very early post-natal variables appears feasible.

Our results could pave the way for new AI applications in the neonatal field. They would enable risk-adjusted analyses of outcomes, healthcare costs, and management strategies, ultimately improving the overall quality of care.

## Data Availability

Clinical Trial Registration: NCT04609163.
